# Does the audiogram shape influence the intracochlear recording of Electrocochleography during and after cochlear implantation?

**DOI:** 10.3389/fnins.2024.1530216

**Published:** 2025-01-16

**Authors:** Sabine Haumann, Max E. Timm, Andreas Büchner, Thomas Lenarz, Rolf B. Salcher

**Affiliations:** ^1^Department of Otorhinolaryngology, Hannover Medical School, Hanover, Germany; ^2^Cluster of Excellence "Hearing4all", Hanover, Germany

**Keywords:** cochlear implant, hearing preservation, CI electrode location, electrocochleography, cochlear monitoring

## Abstract

During cochlear implant (CI) surgery, it is desirable to perform intraoperative measurements such as Electrocochleography (ECochG) to monitor the inner ear function and thereby to support the preservation of residual hearing. However, a significant challenge arises as the recording location of intracochlear ECochG via the CI electrode changes during electrode insertion. This study aimed to investigate the relationships between intracochlear ECochG recordings, the position of the recording contact within the cochlea relative to its anatomy, and the implications for frequency and residual hearing preservation. Intraoperative ECochG recordings were conducted using the CI electrode (MED-EL) during the insertion of hearing preservation electrodes and after the insertion process. Recordings were continuously conducted using the most apical electrode (contact 1) during insertion. After insertion, the recordings were performed on all different electrode contacts. The electrode location in the cochlea during insertion was estimated using mathematical models and preoperative clinical imaging, while the postoperative electrode position was determined using postoperative clinical imaging. The study involved 10 adult CI recipients. In those with good low-frequency hearing, an increase in signal amplitude was observed, with the highest amplitudes closest to the stimulation frequency generators, and no phase change was observed. Conversely, patients with flat hearing loss exhibited a second peak with an opposite phase in the medial area of the cochlea. This study is the first to suggest that the pattern of the preoperative audiogram may influence the ECochG outcomes measured intraoperatively. Specifically, the ECochG responses during insertion appeared to behave as expected with good low-frequency hearing, while with flat hearing loss there appear to be further effects. These findings indicate that this approach can provide valuable information for the interpretation of intracochlearly recorded ECochG signals.

## Introduction

For the past several years, patients with significant residual hearing have been recommended cochlear implantation (CI) surgery with hearing preservation techniques if satisfactory speech understanding cannot be achieved with a conventional hearing aid. This concept primarily aims to provide these patients with a combination of acoustic and electrical hearing (James et al., 2005; Gantz et al., 2005; Baumgartner et al., 2007; Lenarz et al., 2009; Helbig et al., 2011; Von Ilberg et al., 2011; Incerti et al., 2013; Büchner et al., 2017; Roland et al., 2018; Gantz et al., 2018). For this purpose, monitoring of the cochlear function during CI electrode insertion is desirable to support the preservation of residual hearing.

An electrophysiologic method that has been increasingly used for monitoring the functional status of the cochlea during electrode insertion is electrocochleography (ECochG); for reviews, see, for example Kim (2020), Barnes et al. (2021), Kim (2024). In this method, the acoustically evoked electrical activities in the inner ear can be recorded intraoperatively during the insertion of the electrode.

The recorded ECochG signal comprises several components generated by the hair cells, basilar membrane, and auditory nerve, which are described thoroughly in the literature, e.g., Eggermont (1974), Snyder and Schreiner (1984), Forgues et al. (2014), Buechner et al. (2022), Haumann et al. (2024). Most studies concentrate on cochlear microphonics (CMs) and auditory nerve neurophonics (ANNs). These components are difficult to separate and are often referred to as ongoing responses (ORs). These components best reflect the status of the hair cells and the auditory nerve in the low frequencies, which is the range where most CI candidates have significant residual hearing.

The acoustic stimulus for ECochG is usually delivered via an insert earphone placed in the outer ear canal. The response signal can be recorded using either extracochlear or intracochlear recording electrodes.

Many research groups have found some correlations between extracochlear recordings made at or near the promontory wall and the preservation of residual preservation (Radeloff et al., 2012; Fitzpatrick et al., 2014; Adunka et al., 2016; Dalbert et al., 2016; Dalbert et al., 2018) or with postoperative speech perception (Abbas et al., 2017; Walia et al., 2022). However, there has been a mixed picture of the overall benefit relative to the effort required for extracochlear recording (Haumann et al., 2024). As such, intracochlear measurements have become routine for cochlear monitoring. Here, the signal can be recorded via the CI electrode, and all CI manufacturers now offer routine implementations (Dalbert et al., 2021; Buechner et al., 2022; O’Leary et al., 2023; Saoji et al., 2023; Sijgers et al., 2023; Greisiger et al., 2024; Haumann et al., 2024; Panario et al., 2024; Walia et al., 2024).

Potentials recorded with intracochlear electrodes are larger and are presumably more sensitive because the signal is recorded much closer to the generator than with extracochlear measurements. The main drawback of this approach is that the recording site moves during insertion, so several factors besides trauma to the cochlea contribute to the temporal dynamics of the signal, such as the changing distance between the recording electrode and the generator of the response. In addition, temporary blockages of the basilar membrane can reduce the signal amplitude.

Currently, several research groups are investigating the dynamics of the recorded signal during CI electrode insertion and their relationship to the preservation of residual hearing. Ideally, the signal recorded with the most apical electrode should continuously increase during insertion as the generator’s characteristic frequency range, which depends on the stimulation frequency used, is approached. Depending on the electrode length, the electrode can be inserted beyond this characteristic frequency range, which should result in an amplitude drop on the most apical contacts. To analyze this, Saoi et al. stimulated with different frequencies during insertion to investigate the amplitude behavior when characteristic frequency ranges of generators are crossed in any case (Saoji et al., 2019; Saoji et al., 2023). They observed the expected amplitude drop under these conditions.

Haumann et al. differentiated whether a drop in amplitude during insertion could be explained by a change in the electrode position or by trauma to the cochlea based on the postoperative imaging and modeling of the exact location of the characteristic frequency region. After insertion, ECochG was measured again via various CI contacts (Haumann et al., 2024). Other research groups hypothesized that a drop in the first half of insertion is more likely due to transient events such as temporary contact of the array with the basilar membrane, and a drop in the second half of insertion is more consistent with trauma and subsequent residual hearing loss (Koka et al., 2018; Sijgers et al., 2021).

Other groups have examined the phase and latency of the signal along with the amplitude. The hypothesis is that a drop in amplitude accompanied by a phase shift is not critical since the signal’s phase also changes when the electrode is advanced. Conversely, an amplitude drop without an accompanying phase change would suggest the presence of cochlear trauma. It was found that the latter correlated quite strongly with residual hearing loss (Buechner et al., 2022). Other research groups have also suggested including phase, latency, and neuronal components in the interpretation of these signals (Koka et al., 2018; Giardina et al., 2019; Sijgers et al., 2021). Greisiger et al. combined the recording of intracochlear ECochG with fluoroscopy and analyzed the behavior of the recorded signal together with the microscopy video and the postoperative CT scan (Greisiger et al., 2024). The main aim was to identify critical steps during CI electrode insertion, and they postulated that the combination of fluoroscopy and ECochG provides helpful information in this regard.

Another research group measured ECochG on several contacts after electrode insertion and calculated the individual components from the signal. In a large patient group, they observed that CMs and SPs, in particular, explain a moderate part of the variance in later, residual hearing and CI-aided speech understanding (Panario et al., 2024).

In summary, intracochlear ECochG appears to have utility for monitoring residual hearing preservation, but there are still some unanswered questions about the behavior of the measured signal and subsequent residual hearing preservation.

In this study, intracochlear ECochG was measured in ten adult patients during and after CI insertion, and, together with pre- and postoperative imaging, the relation to subsequent residual hearing preservation was examined. Particular attention was paid to the course of the preoperative hearing threshold, as a corresponding influence was indicated in an earlier study (Haumann et al., 2024). In the present study, more measurement points could be recorded due to the continuous measurement during insertion and the recording at 12 electrodes after insertion. This allows for a more precise evaluation than in the prior study. In addition, the phase of the signal and the course of the CI electrode impedances were analyzed in the present study.

## Methods and materials

In this work, a monocentric, prospective study is presented. The study was approved by the local ethics committee (approval number 10007_BO_S_2021) and is in accordance with the ethical standards of the Declaration of Helsinki. Ten patients undergoing CI surgery underwent intraoperative intracochlear (IC) ECochG during and after electrode insertion using a research tool provided by the implant manufacturer (MED-EL, Innsbruck, Austria). The recordings were performed continuously during electrode insertion using the most apical CI electrode contact for recording (C1) with a stimulation frequency of 500 Hz. After intraoperative insertion, recordings were performed at all CI electrode contacts using a stimulation frequency of 500 Hz. Relationships were investigated between intraoperative electrophysiologic recordings, the location of the electrode within the cochlea, and the pre- and postoperative pure tone audiogram (PTA) thresholds on audiograms.

### Preoperative evaluation

The preoperative CI candidate evaluation at our clinic follows a standardized protocol (Haumann et al., 2019). This protocol includes both subjective and objective audiometric evaluations, clinical imaging such as CT and fMRI scans, and other relevant examinations. Patients select an implant system based on their preferences. The appropriate electrode length was chosen by considering residual hearing and the length of the cochlea (Würfel et al., 2014; Timm et al., 2018).

Pure tone audiometry (PTA) was performed with an AD2117 audiometer from Audio-DATA GmbH (Duvensee, Germany), calibrated to hearing level (HL). Air conduction testing was performed using HDA300 headphones from Sennheiser Electronic GmbH (Wedemark, Germany), and bone conduction testing was performed with a KLH96 bone transducer from Westra Elektroakustik GmbH (Meitingen, Germany).

### Intracochlear ECochG recordings using the CI electrode

#### Measurement setup

For IC recordings, different MED-EL (Innsbruck, Austria) FLEX electrode arrays were used with the clinical CI measurement setup for MED-EL implants (MAX-Box, coil, and laptop) and the manufacturer’s research software (MAESTRO 9.0.3, MED-EL). The acoustic stimulation was delivered via a Dataman 531 Arbitrary Waveform Generator (Dataman, Maiden Newton, United Kingdom) triggered by the MAESTRO Software. Stimulation was delivered using insert earphones (ER-3C 50 Ohm, Etymotic, Fort Worth, Texas, USA) with a sterile foam plug placed in the outer ear canal.

A 500 Hz Hamming windowed pure tone burst of 8 ms duration was used for stimulation. The stimulation level was calibrated to hearing level (HL) and set intraoperatively to 110 dB. The polarity of the stimulus was set to condensation.

The following parameters were chosen for the recordings: An 8.1 ms recording window was used to capture the complete wave for better signal processing analysis. There was a 2 ms measurement delay and no trigger delay. The measurement was carried out for continuous recordings until the electrode was inserted and the cable was fixed. There were n = 130 iterations for sequential recordings, which were averaged for each recording electrode, yielding a total recording time of 4.5 min for all 12 electrodes.

#### Intra- and postoperative procedures

The CI implantation was performed according to our standards (Lenarz et al., 2022). The CI electrode insertion was performed manually or robot-assisted with RobOtol^®^ (Collin Medical, Bagneux, France). During the CI electrode insertion, IC potentials were recorded continuously using the most apical electrode contact (C1). The insertion progress, measured by the number of electrodes inserted, was determined visually from the video recording. Partial insertion was sometimes used (Lenarz et al., 2019), so the final number of inserted electrodes was not always 12. Partial insertion aims to preserve residual hearing by mimicking a short electrode, while in the event of later degeneration of the residual hearing, the electrode can be fully inserted, and the patient can receive electrical stimulation over the full frequency range.

After insertion, a sequential recording was performed using all electrode contacts separately. The final position of the electrode was evaluated per clinical routine via cone beam CT scan, intraoperatively or one day postoperatively. The location of the electrode within the cochlea was determined using the pre- and postoperative images, and the characteristic frequency region was estimated as described by Haumann et al. (2024). In short, the cochlear lateral wall (LW) was tracked using OsiriX MD (Pixmeo SARL, Switzerland), yielding the 3D spiral shape of the cochlea. The full reconstruction of the scala tympani (ST) was performed by combining the 3D shape of the LW with cross sections of the ST derived from micro CT imaging, as is explained in detail in Schurzig et al. (2023). The path of the electrode array inside the ST was estimated using the average distance between LW and the electrode array, which depends on the array and the insertion angle. The electrode array was virtually inserted so that the specified number of electrodes were located inside the cochlea. The final location of the array in the cochlea after insertion was segmented from the postoperative imaging and registered to the LW using the HelReg method (Schurzig et al., 2018), and the tonotopic frequencies were allocated based on an organ of Corti frequency mapping approach (Helpard et al., 2021).

The impedances of the intracochlear electrodes and the ground path were recorded per clinical routine using the clinical CI measurement setup by MED-EL. These were evaluated intraoperatively after CI electrode insertion, at the test tone (1–3 days after surgery), and at the first fitting, which took place 5 weeks after surgery.

### Data analysis

#### PTA thresholds

The differences in the low-frequency air conduction thresholds before surgery and at the first fitting appointment were tested for significance at a 5% level with a paired-sample T-test, and the correlation coefficients were calculated using the respective MATLAB functions. Hearing preservation for the different inserted electrode depths (IED) was classified as similar to Suhling et al. (2016). The audiometric pure tone low-frequency hearing threshold (PTA_low_) was calculated as the mean of the air conduction thresholds at 250, 500, and 1000 Hz. If no response was observed at the highest stimulated level, the response was set to 10 dB above the audiometer limit (105 dB HL for 250 Hz, 110 dB HL for 500 Hz, and 110 dB HL for 1000 Hz). The postoperative hearing preservation was classified into three levels according to the pre- to postoperative PTA_low_ shift: a shift of up to 15 dB, a shift between 15 dB and 30 dB, and a shift of more than 30 dB.

#### ECochG recordings

ECochG recordings were averaged from 100 repetitions of the same stimulus. The averaged recordings were then band-pass filtered between 30 Hz and 10 kHz using a zero-phase, forward-backward second-order Butterworth filter before analysis. Aside from this, no other processing has been conducted. Since all recordings were the same length, the frequency bandwidth for FFT estimation was also consistent. The noise floor for each session was estimated to be the mean amplitudes of three bins on the left and three bins on the right of the stimulus frequency. A recording was considered a response if its amplitude was at least three standard deviations above this noise floor. The entire insertion process was considered one session for continuous recordings performed during insertion. All recordings made under one task were considered one session for sequential, post-insertion measurement.

### Subject demographics

A total of 10 patients (seven male) with low-frequency residual hearing participated in the study. The mean age was 56 years (18–77 years). Implantation was performed on the right side in six cases. All received a MED-EL Synchrony 2 implant with different electrode lengths (Flex28 in nine cases and FlexSoft in one case) and different IEDs. Their details are given in Table 1.

**Table 1:** Details of the subjects and the implanted electrodes.

ID	Electrode	Inserted electrode depth (mm)	Age at surgery (yrs)	Sex	Side	Duration of hearing impairment (yrs)	Etiology
S01	Flex28	20.9	51.8	f	r	19	unknown/progressive
S02	Flex28	27.1	76.9	m	l	8	unknown/progressive
S03	Flex28	22.3	34.9	f	l	32	unknown/progressive
S04	Flex28	28.6	18.2	m	r	16	unknown/progressive
S05	Flex28	25.6	67.4	m	l	30	sudden hearing loss/progressive
S06	Flex28	28.0	36.9	m	l	27	sudden hearing loss
S07	Flex28	26.9	61.3	m	r	24	unknown
S08	Flex28	25.9	59.7	f	r	7	sudden hearing loss/progressive
S09	Flex28	26.2	76.7	m	r	71	unknown
S10	FlexSoft	26.7	72.2	m	r	66	unknown/progressive

## Results

### Hearing preservation as measured by pure tone audiometry

**Figure 1:**
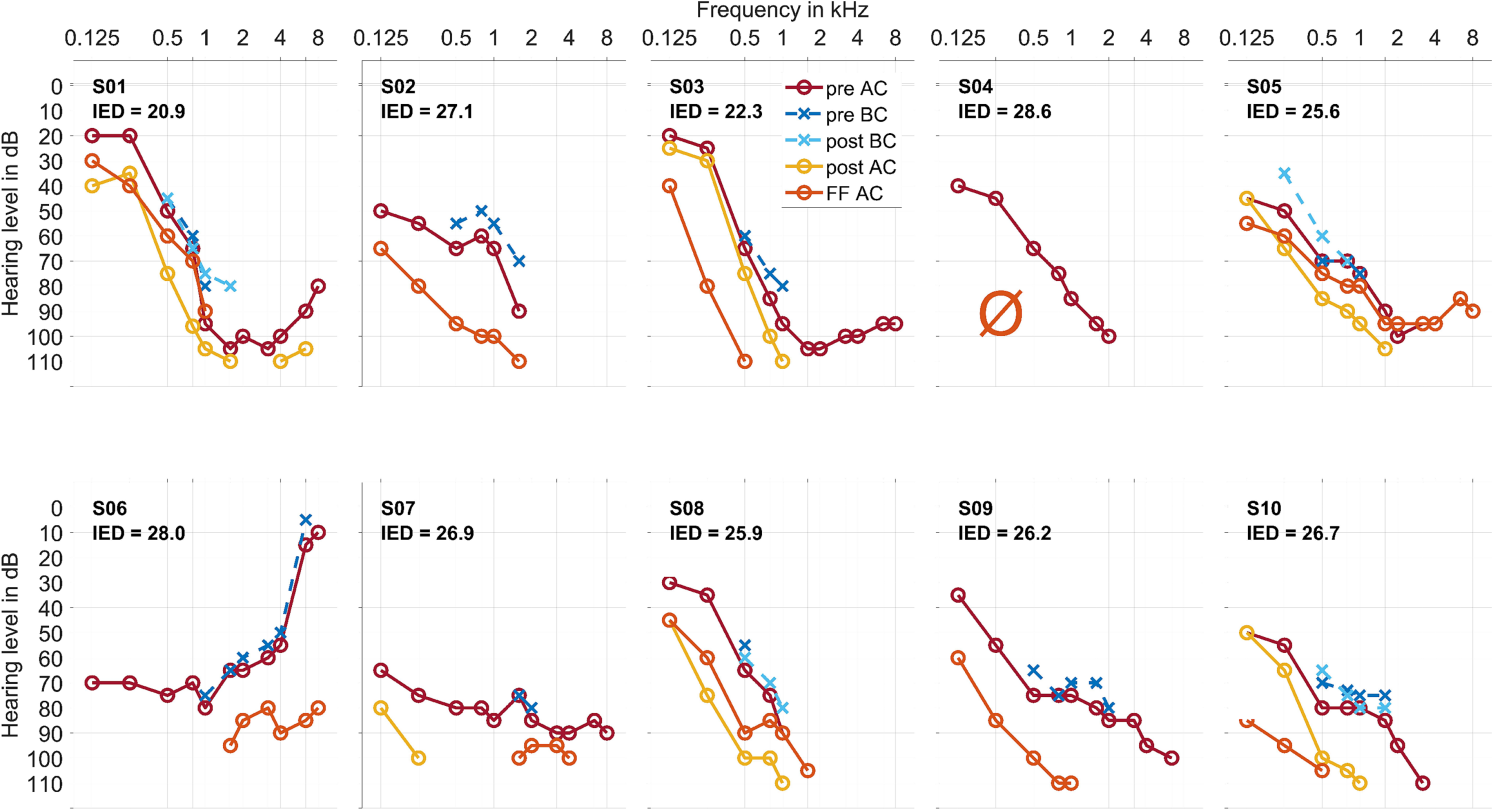
Air conduction thresholds (AC, in warm coloring, with solid lines and circles) and bone conduction thresholds (BC, in cold coloring, with dashed lines and crosses) for each patient at the preoperative, postoperative, and first fitting (FF) measurement time points. The Inserted Electrode Depth (IED, given in mm) for each patient is also shown. For S03 and S10, the preoperative data point at 750 Hz was interpolated visually. In S02, S06, and S09, there was no measurable hearing 1–3 days postoperatively. In S04, there was no measurable hearing postoperatively or at the first fitting measurement time point.

The audiograms of all 10 individuals are given in Figure 1. The hearing preservation classification as measured by PTA is given in Table 2. Preoperative tympanograms for all 10 cases were unremarkable, showing no evidence of middle ear fluid or negative pressure.

**Table 2:** Hearing preservation classified by low tone air conduction threshold shifts before surgery and at the first fitting appointment, usually 5 weeks after surgery, similar to Suhling et al. (2016).

	PTA_low, first fit_ – PTA_low, pre_		
	∆ PTA_low_ ≤ 15 dB	15 dB < ∆ PTA_low_ ≤ 30 dB	∆ PTA_low_ > 30 dB
IED ≤ 24 *n* = 2	1 50%	0 0%	1 50%
IED > 24 *n* = 8	1 12.5%	3 37.5%	4 50%

The low-frequency PTA was calculated as the mean of the threshold at 250, 500, and 1000 Hz.

Table 3 details the individual audiometric threshold shifts from pre- to post-surgery. The differences in the low-frequency air conduction thresholds before surgery andat the first fitting appointment were significant, with a correlation coefficient of *r* = 0.74*.

**Table 3:** Inserted electrode depth (IED), audiometric thresholds PTA _low_ before surgery and at FF, and hearing preservation group (HP group) classified by audiometric threshold shifts according to Suhling et al. (2016).

ID	IED (mm)	PTA_low_ (dB HL) before surgery	Preop transtymp ECochG threshold (dB nHL)		PTA_low_ (dB HL) at first fitting appointment and HP group	
			CM 2 kHz	CAP click	PTA_low_ (dB HL) 0.25–1 kHz	HP group
S01	20.9	55.0	70	nr	63.3	0–15 dB
S02	27.1	61.7	not possible (medical)		91.7	15–30 dB
S03	22.3	61.7	80	nr	103.3	>30 dB
S04	28.6	65.0	90	nr	118.3	>30 dB
S05	25.6	65.0	60	nr	71.7	0–15 dB
S06	28.0	75.0	60	60	118.3	>30 dB
S07	26.9	80.0	80	nr	118.3	>30 dB
S08	25.9	63.3	70	nr	80.0	15–30 dB
S09	26.2	68.3	80	nr	98.3	15–30 dB
S10	26.7	71.7	declined by patient		106.7	>30 dB

nr - no responses.

### Impedances of the CI electrode contacts

**Figure 2:**
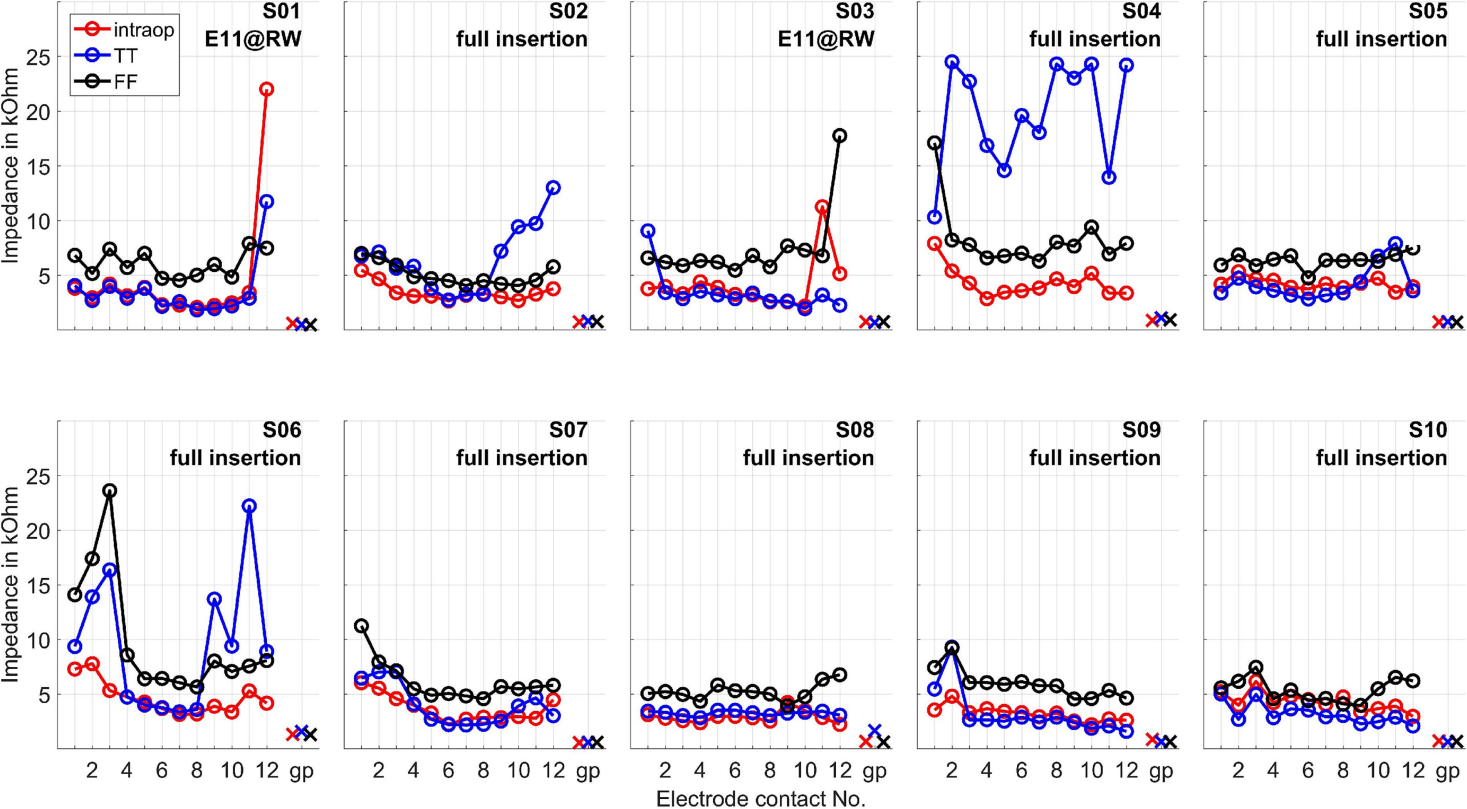
Impedances of the CI electrode contacts measured intraoperatively (intraop), at test tone (TT, 1–3 days after surgery), and at first fitting week (FF, 5 weeks after surgery). The impedances are given for all electrode contacts and the ground path (gp). E11@RW means a partial insertion such that the electrode was intentionally inserted partially until electrode contact 11 was positioned at the round window.

The impedances of the CI electrode contacts are given in Figure 2.

### Intracochlearly recorded ongoing responses: example cases

**Figure 3:**
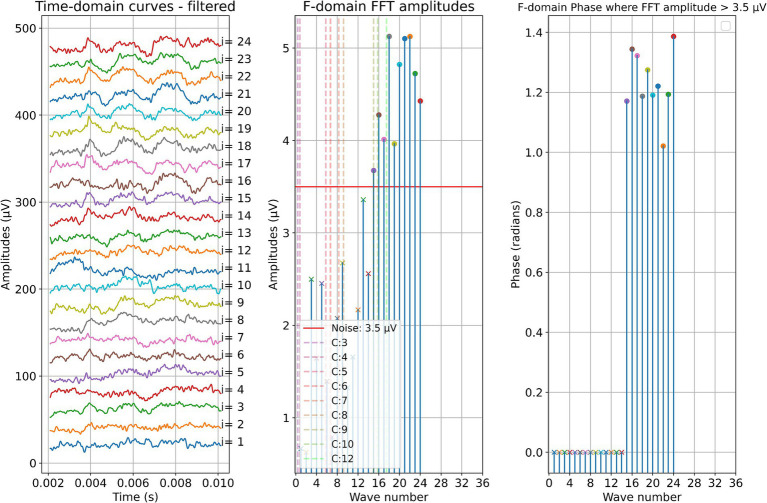
ECochG measurements performed during insertion in subject 1. Recordings were performed with contact 1. The left panel shows the data in the time domain, with higher wave numbers corresponding to greater electrode insertion depths. The middle panel shows the amplitudes of the wave number in terms of the FFT bin at 500 Hz. The red line represents the noise floor, and the amplitude markers are filled dots if the amplitude exceeds the noise level and crosses if the amplitude is lower than the noise level. The colored dashed lines mark the number of inserted electrodes. On the right panel, the response phase is illustrated for the waves with amplitude exceeding the noise level.

**Figure 4:**
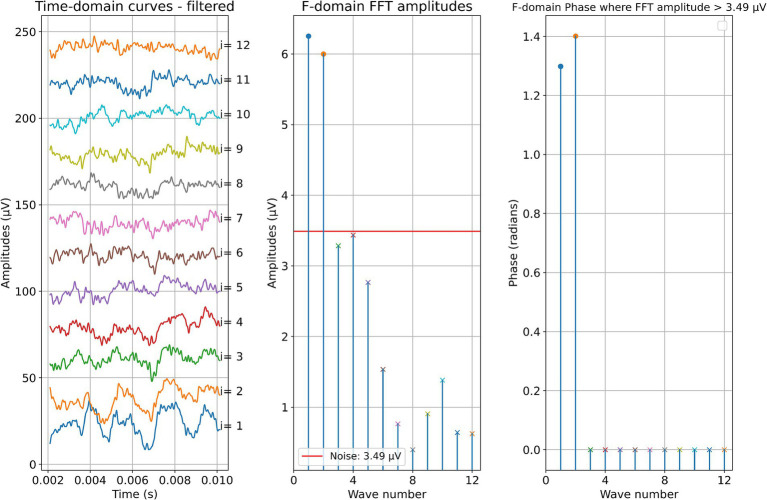
Post-insertion ECochG measurements in subject 1. The left panel shows the data in the time domain, with the number on the right side indicating the recording contact. The middle panel shows the amplitudes of the wave number in terms of the FFT bin at 500 Hz. The red line represents the noise floor, and the amplitude markers are filled dots if the amplitude exceeds the noise level and crosses if the amplitude is lower than the noise level. The wave number corresponds to the recording contact. On the right panel, the response phase is illustrated for the waves with amplitude exceeding the noise level.

**Figure 5:**
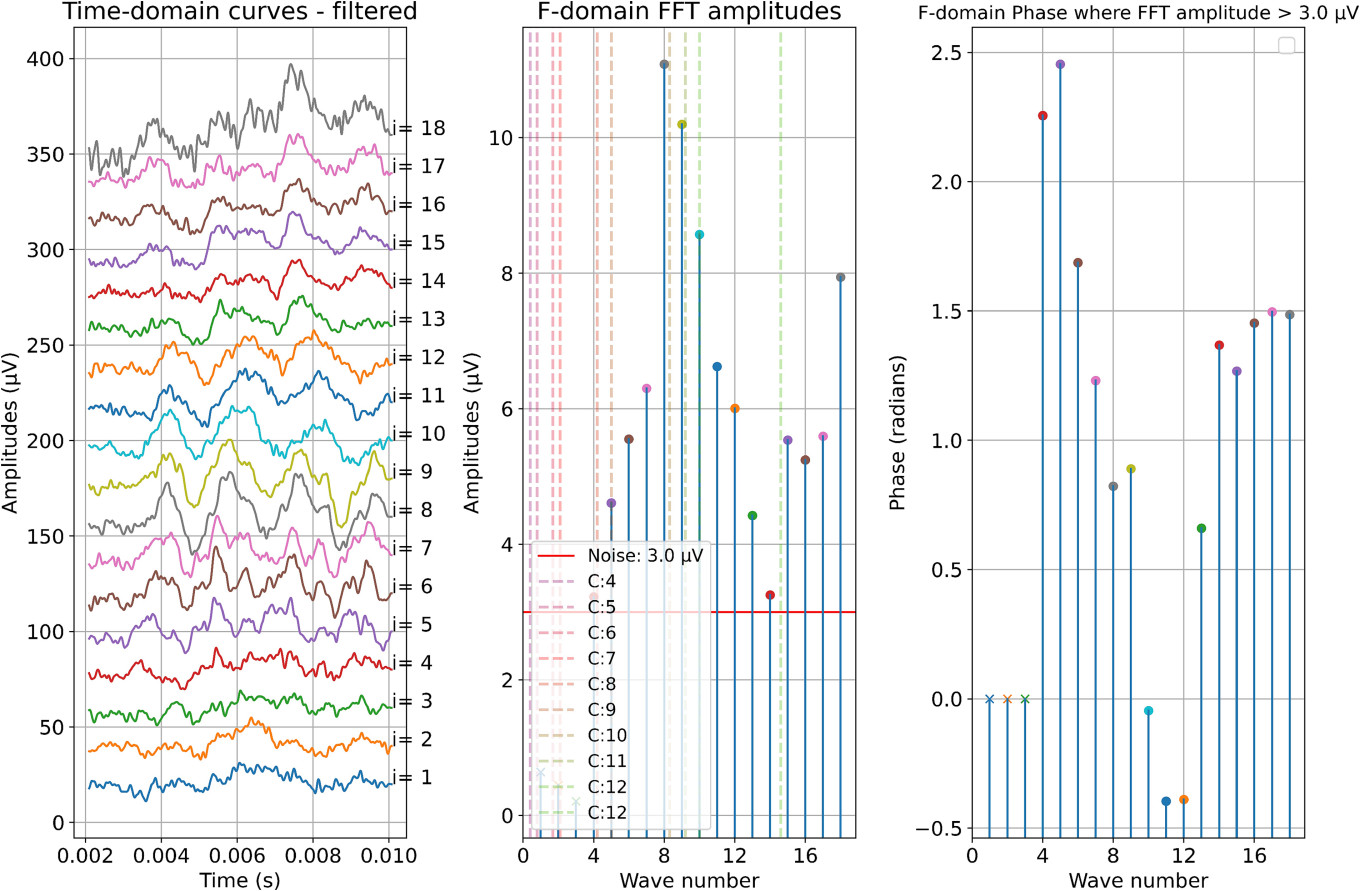
ECochG measurements performed during insertion in subject 5. Recordings were performed with contact 1. The left panel shows the data in the time domain, with higher wave numbers corresponding to greater electrode insertion depths. The middle panel shows the amplitudes of the wave number in terms of the FFT bin at 500 Hz. The red line represents the noise floor, and the amplitude markers are filled dots if the amplitude exceeds the noise level and crosses if the amplitude is lower than the noise level. The colored dashed lines mark the number of inserted electrodes. On the right panel, the response phase is illustrated for the waves with amplitude exceeding the noise level.

**Figure 6:**
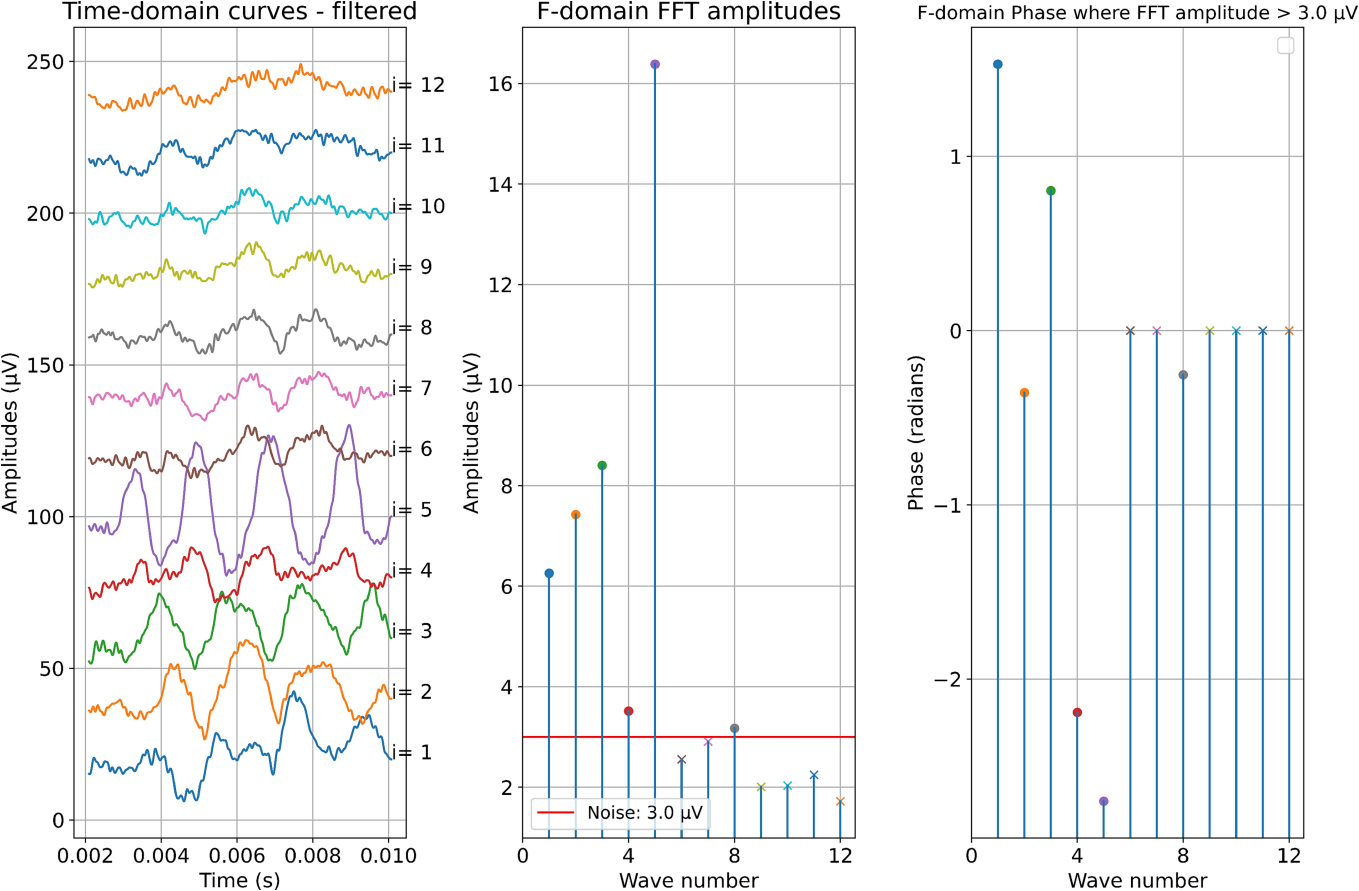
Post-insertion ECochG measurements in subject 5. The left panel shows the data in the time domain, with the number on the right side indicating the recording contact. The middle panel shows the amplitudes of the wave number in terms of the FFT bin at 500 Hz. The red line represents the noise floor, and the amplitude markers are filled dots if the amplitude exceeds the noise level and crosses if the amplitude is lower than the noise level. The wave number corresponds to the recording contact. On the right panel, the response phase is illustrated for the waves with amplitude exceeding the noise level.

Two example cases are given in detail in this section. For subject 1, Figure 3 shows the measurements recorded during electrode insertion, and Figure 4 shows the measurements recorded after electrode insertion. For subject 5, Figure 5 shows the measurements recorded during electrode insertion, and Figure 6 shows the measurements recorded after electrode insertion.

### Intracochlearly recorded ongoing responses: all data

**Figure 7:**
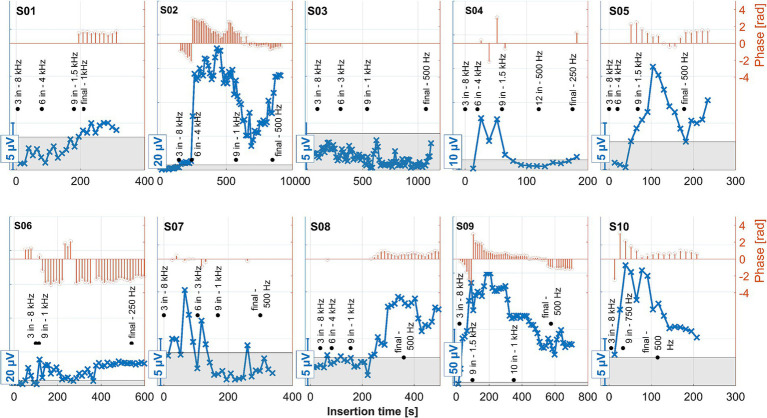
ECochG recordings performed during insertion for all 10 subjects. Response amplitudes as a function of insertion time are shown in blue. As the response amplitudes differed between subjects, different scales were chosen for the *y*-axes of each panel. These are indicated by the bars at the bottom left of each panel. The orange stem plots at the top of each panel show the signal’s phase. The scaling is indicated in the second *y*-axis to the right. Phase data only show where the amplitude exceeded the noise level (the gray area within each panel). The recordings were performed with the apical-most contact (C1), and the stimulation frequency was 500 Hz. The black annotations show the number of inserted contacts at various representative locations, together with the model-based estimated frequency of the organ of Corti where contact 1 was currently located at that time point. The estimated frequency was rounded to the nearest common frequency for audiometric testing.

**Figure 8:**
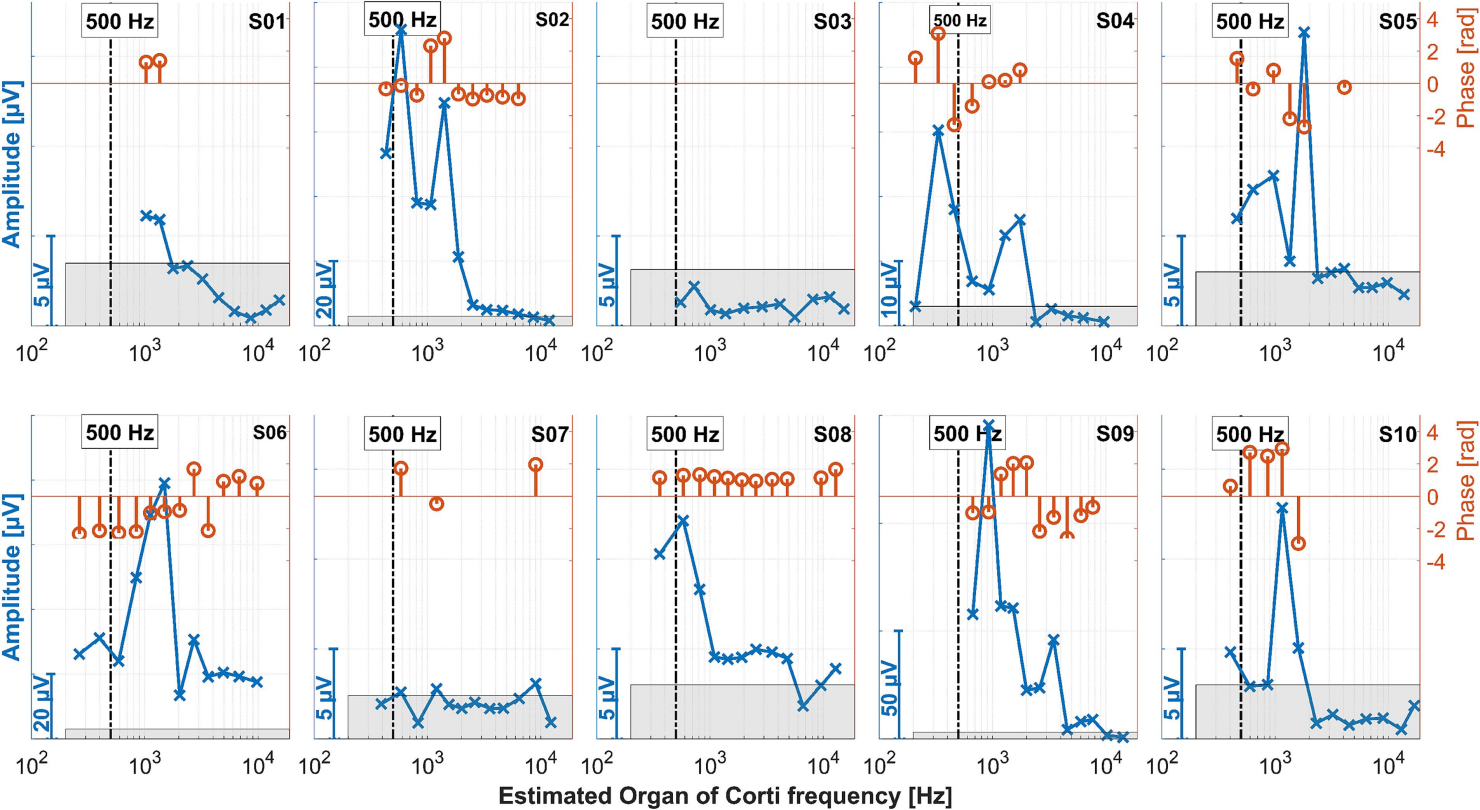
ECochG recordings performed after insertion for all 10 subjects. The amplitudes in the time domain for all different electrode contacts are shown in blue. The *x*-axis gives the location of the recording contact and the estimated frequency of the organ of Corti modeled from the postoperative neuroimaging. The vertical bars represent the stimulation frequency of 500 Hz. Since the response amplitudes differed between patients, different scales were chosen for the y-axis of each panel, as indicated by the bars at the bottom left of each panel. The same *y*-axis scales were used as in Figure 7. The orange stem plots at the top of each panel show the signal phase; the scaling is indicated in the second *y*-axis on the right. The phases only show where the amplitude exceeded the noise level (the gray area within each panel).

The measurements for all patients during electrode insertion are given in Figure 7, and those after insertion are given in Figure 8.

## Discussion

In this study, intracochlear ECochG responses were recorded intraoperatively during and after CI electrode insertion in 10 subjects. The electrode position in the cochlea was estimated together with the presumed frequency generator for each patient. Intraoperative recordings were compared to pre- and postoperative PTA thresholds. In an earlier study, intracochlear ECochG recordings were made at specific insertion steps during electrode insertion. After insertion and 6 months postoperatively, intracochlear ECochG was recorded at a subset of electrode contacts distributed throughout the cochlea (Haumann et al., 2024). In the present study, the resolution of the recordings was improved, as the new study tool of MED-EL allows for continuous recordings during CI electrode insertion. Moreover, all CI electrode contacts were used for the recordings after insertion because this measurement was faster than the former one.

Preservation of residual hearing in the low-frequency range with air conduction was classified into three groups similar to Suhling et al. (2016). At the first fitting week (usually 5 weeks after surgery), one subject with an IED of ≤24 mm was classified within the best hearing preservation group (0–15 dB), and one to the worst group (>30 dB). For the eight subjects with an IED of >24 mm, one subject was classified to the best group, three to the medium group (15–30 dB), and four to the worst group (>30 dB). One of these subjects (S04) also experienced a complete loss of residual hearing. These results are in line with the findings of Suhling et al. (2016). With two subjects (S02 and S03), the CI electrode insertion was performed with robotic assistance using RobOtol^®^. This method allows for extremely slow insertion speeds, as it was previously assumed that slow insertion was generally better for residual hearing preservation. This could not be demonstrated in our data, which is also consistent with recent findings on the behavior of the CI electrode in fluids with a similar viscosity to perilymph (Fröhlich et al., 2024; Fröhlich et al., 2024).

All intraoperative measurements have the disadvantage that they can only be used to investigate processes that occur up to the end of the CI surgery. It is known that implantation can also induce postoperative processes such as inflammation (Seyyedi and Nadol, 2014), neo-ossification and fibrosis around the implant (Quesnel et al., 2016), and apical degeneration of the stria (O'Malley et al., 2024).

Tan et al. (2024) observed that significant increases in CI electrode impedance were associated with residual hearing loss, suggesting that such impedance elevation could be due to postoperative inflammation. This phenomenon was evident in our observations for subjects S04 and S06, both of whom belonged to the group with the poorest preservation of residual hearing. These were also the two cases with the deepest electrode insertions.

The ECochG measurement for subject S04 showed a notable decrease in amplitude when the surgeon adjusted the grip on the insertion forceps toward the end of the electrode placement (Figure 7). Under typical circumstances, such a significant drop in amplitude might suggest that no further stimulus responses would be recorded post-insertion. Contrary to expectations, however, the ongoing response (OR) amplitudes recorded after the insertion were actually larger (Figure 8).

On the other hand, when the amplitude drop occurred during insertion, the frequency generator was crossed according to the calculations (see discussion point below). However, the residual hearing was lost postoperatively in this subject and has not recovered. Therefore, changing the grip of the insertion forceps could also have triggered a postoperative degeneration process, which would be reflected in the increased impedance of the CI electrode. Also, in patient S06, good stimulus responses were measured at the end and after the insertion.

No strong increase in impedances was observed in the other patients. Thus, our study did not observe an increase in impedance without residual hearing loss. For patients who experienced residual hearing loss without an increase in impedance (S03, S07, and S10), this could indicate that the residual hearing was already impacted during the operation (rather than by postoperative inflammation). In patient S07 OR responses were detected at the beginning of the insertion, although they were quite small. In the second half of the insertion, these disappeared and recovered only marginally after insertion, with the residual hearing deteriorating after surgery. This finding is in line with the hypothesis that a drop in response amplitudes during the second half of insertion could indicate trauma and subsequent residual hearing loss (Koka et al., 2018; Sijgers et al., 2021). In patient S03, no responses above the noise level were detected by the automatic algorithm from the beginning of the measurement. Visually, responses could be observed, but these were noisy and of small amplitude. On this basis, it is possible that the residual hearing of this patient was impaired quite at the beginning of the CI electrode insertion. The amplitude behavior in patient S10 does not fully explain the deterioration in residual hearing, as the amplitude had fallen at the end of the insertion. However, responses were still clearly detectable and were also present postoperatively in a similar amplitude range to during the insertion. In S02, an impedance increase in the basal area of the cochlea was observed postoperatively, but this was lower than in S04 and S06. The residual hearing had also deteriorated significantly in this patient. However, the residual hearing, for example, in S10, also deteriorated significantly by the first fitting without a strong impedance increase. As such, the selectivity of this measure would have to be checked on a larger group of patients. It is also possible that the impedances increased between the test tone and the first fitting, so further measurement time points may need to be scheduled here in future studies.

Previous publications have already described some of the uncertainties of ECochG recordings. For example, ORs can only reflect the status of the inner ear, while the auditory pathway includes more structures (Fitzpatrick et al., 2014; Campbell et al., 2015; Haumann et al., 2019). The different times of the measurements should also be taken into account. Intraoperative measurements can only represent the status at the end of the operation, while the first degenerative processes have already occurred by the time of the postoperative hearing test. Nevertheless, several studies show a good correlation between intracochlearly recorded ORs and audiometric threshold when measured postoperatively on the same day (Campbell et al., 2015; Dalbert et al., 2015; Koka et al., 2017; Haumann et al., 2019; Krüger et al., 2020; Buechner et al., 2022). It can, therefore, be assumed that intracochlearly recorded ORs at the end of the surgery reflect the status of residual hearing with clinically acceptable accuracy.

One limitation of intracochlear OR recordings, compared to other methods under investigation, is the uncertainty introduced by changes in the recording location during CI electrode insertion. Currently, it is not fully understood how the amplitude ideally should behave. In theory, the amplitude should rise with ongoing insertion. An amplitude drop can have various causes. For example, a transient drop could be caused by the electrode mechanically blocking or dampening the basilar membrane. Also, the recording electrode may be inserted beyond the characteristic frequency region of the stimulus (Haumann et al., 2019; Buechner et al., 2022; Saoji et al., 2023). In both cases, residual hearing should not be affected.

In contrast, acute trauma to the cochlea should be accompanied by a loss of residual hearing. In an earlier study, the location of the electrode in the cochlea was calculated using clinical neuroimaging, and the corresponding frequency was estimated to determine whether the frequency generator had been passed by the recording electrode (Haumann et al., 2024). However, when those experiments were conducted, the clinical MED-EL software could only perform these measurements during discrete insertion steps, so the grid was somewhat coarse. The post-insertion measurement, on the other hand, is purely static, so by comparing the two measurements during and after insertion, it can be distinguished with a certain degree of reliability whether a signal drop is presumably an effect of the changing distance to the generator or whether it could represent an acute trauma. With the MED-EL research software used here, measurements can be carried out continuously during the insertion, and the static measurement post-insertion is also faster so that measurements can be carried out on all 12 contacts.

Because more patients with deeper insertions were included in the current study than in the earlier work, the frequency generator was presumed to be crossed in almost all cases. According to the calculation, the region was not reached in S01 and S09. With S03, the region was presumably reached but not crossed.

In the earlier work, it was already noticeable that in patients where the low-frequency residual hearing was normal or almost normal and the hearing threshold dropped steeply toward the high frequencies, the OR amplitude increased during the insertion or was at its highest after the insertion near the estimated generator frequency. On the contrary, in patients with an almost flat hearing threshold or at least less residual hearing in the low-frequency range and more residual hearing in the high-frequency range, the course of the OR amplitude could not be assigned so clearly.

This effect can be better investigated with continuous measurements or a finer postoperative resolution, which is now possible with the MED-EL tool. Notably, as observed in the data here and in earlier publications, for subjects S01, S03, and S08, the OR amplitude increases toward the estimated generator frequency and peaks after the insertion. The residual low-frequency hearing is normal or almost normal in these patients, and a steep drop-off occurs toward the higher frequencies. Neither in the time course of the insertion nor in the post-insertion measurements, when comparing the recordings on the individual contacts, could a phase shift be observed in these subjects. In contrast, OR amplitudes were found in the medial region of the cochlea in subjects S02, S04, S05, S06, and S09 both during and after insertion. The signal then dropped again to form another peak at or near the expected frequency range. Subject S07 had a second peak in the medial area during insertion, but the signal dropped after insertion, so in the post-insertion recordings, no peaks could be detected. Subject S10 had a peak in the medial region during and after insertion, but there was only a very small peak in the expected frequency range.

The preoperative low-frequency residual hearing was impaired in all the subjects with an OR peak in the medial area, and the audiogram curve was close to flat. There was a phase shift between the peaks in the expected and medial regions of the cochlea of 180 degrees in most of the mentioned subjects. In subject S09, there was also a large phase shift, but here, it occurred between electrode contacts 2 and 3/4. According to the hypothesis that a drop in the first half of insertion is more likely to reflect transient events such as temporary blockage of the basilar membrane, and a drop in the second half of insertion is more consistent with trauma and subsequent residual hearing loss (Koka et al., 2018; Sijgers et al., 2021), the peak in the medial region should not correspond to a trauma to the cochlea. In our data, there was also no correlation with later hearing preservation.

The peak in the expected region in the described patients also missed the estimated area. It is known that at high stimulation intensities, the position of maximum stimulation of the basilar membrane is shifted (Honrubia and Ward, 1968; Robles and Ruggero, 2001). This effect can be an octave or even more. However, in the present study, stimulation was performed at the same level, and in the patients with good low-frequency residual hearing, the CF region was covered quite well. As such, this effect cannot satisfactorily explain the differences in our data. What was noticeable in patients with more flat hearing loss is that they probably had more surviving hair cells distributed across the cochlea, while patients with good low-frequency residual hearing and a steep drop-off in the high-frequency range had significantly less residual hearing and, therefore, probably fewer surviving hair cells in the medium or high-frequency range. It is known that more hair cells contribute to ORs (or their CM component) than just those in the area of the response generator (Cheatham et al., 2011). It could also be possible that surviving hair cells in the mid-range and high-frequency range contribute to the responses, while in patients with a steep drop-off, hair cells contribute to the stimulus responses mainly in the coarse range of the frequency generator. The travel times of the traveling wave on the basilar membrane could explain the differences in phase between the two peaks, and the interference between both responses could explain the amplitude drop between them. However, based on this theory, one would expect the second peak to be wider (as more hair cells should contribute to the response). This would also need to be further investigated.

A limitation of this study is that stimulation was only performed with a stimulation frequency of 500 Hz. At the time of this study, stimulation could only be performed at one frequency during insertion. Here, 500 Hz was chosen because many patients have good residual hearing in this frequency range. However, the cochlea can be stimulated much more broadly with a multi-frequency stimulus, and significantly more precise information about the state of the cochlea during electrode insertion could be expected. The new software version from MED-EL allows multi-frequency stimulation with chirps, which is currently being investigated in a new study.

## Conclusion

Our study shows for the first time that the course of the preoperative audiogram seems to influence the course of the ECochG measured intraoperatively. In cases with a typical audiometric threshold for electric-acoustic stimulation with good low-frequency residual hearing, the OR amplitude rose during the insertion. OR amplitudes during and after insertion for the subjects with less preoperative low-frequency hearing showed a second peak in the medial area of the cochlea, which went along with a strong phase shift. Thus, with good low-frequency residual hearing, ECochG curves appear to behave as expected during insertion. In contrast, for those with flat hearing loss there appear to be further effects which need to be investigated in future studies. Based on our new data, we would like to confirm our recommendation to match the position of the electrode in the cochlea with the generator frequency again to rule out a crossing of the generator frequency as the cause of the amplitude drop. Moreover, the course of the preoperative audiogram should be included in the interpretation of the measured amplitude curves.

## Data Availability

The raw data supporting the conclusions of this article will be made available by the authors, without undue reservation.

## References

[ref1] Abbas P. J. Tejani V. D. Scheperle R. A. Brown C. J. (2017). Using neural response telemetry to monitor physiological responses to acoustic stimulation in hybrid cochlear implant users. Ear Hear. 38, 409–425. doi: 10.1097/AUD.0000000000000400, PMID: 28085738 PMC5482777

[ref2] Adunka O. F. Giardina C. K. Formeister E. J. Choudhury B. Buchman C. A. Fitzpatrick D. C. (2016). Round window electrocochleography before and after cochlear implant electrode insertion. Laryngoscope 126, 1193–1200. doi: 10.1002/lary.25602, PMID: 26360623 PMC5949050

[ref3] Barnes J. H. Yin L. X. Saoji A. A. Carlson M. L. (2021). Electrocochleography in cochlear implantation: development, applications, and future directions. World J. Otorhinolaryngol. 7, 94–100. doi: 10.1016/j.wjorl.2020.04.006, PMID: 33997718 PMC8103527

[ref4] Baumgartner W.-D. Jappel A. Morera C. Gstöttner W. Müller J. Kiefer J.. (2007). Outcomes in adults implanted with the FLEX soft electrode. Acta Otolaryngol. 127, 579–586. doi: 10.1080/00016480600987784, PMID: 17503226

[ref5] Büchner A. Illg A. Majdani O. Lenarz T. (2017). Investigation of the effect of cochlear implant electrode length on speech comprehension in quiet and noise compared with the results with users of electro-acoustic-stimulation, a retrospective analysis. PLoS One 12:e0174900. doi: 10.1371/journal.pone.0174900, PMID: 28505158 PMC5432071

[ref6] Buechner A. Bardt M. Haumann S. Geissler G. Salcher R. Lenarz T. (2022). Clinical experiences with intraoperative electrocochleography in cochlear implant recipients and its potential to reduce insertion trauma and improve postoperative hearing preservation. PLoS One 17:e0266077. doi: 10.1371/journal.pone.0266077, PMID: 35452461 PMC9032378

[ref7] Campbell L. Kaicer A. Briggs R. O’Leary S. (2015). Cochlear response telemetry: intracochlear electrocochleography via cochlear implant neural response telemetry pilot study results. Otol. Neurotol. 36, 399–405. doi: 10.1097/MAO.0000000000000678, PMID: 25473960

[ref8] Cheatham M. A. Naik K. Dallos P. (2011). Using the cochlear microphonic as a tool to evaluate cochlear function in mouse models of hearing. J. Assoc. Res. Otolaryngol. 12, 113–125. doi: 10.1007/s10162-010-0240-5, PMID: 20957507 PMC3015034

[ref9] Dalbert A. Huber A. Veraguth D. Roosli C. Pfiffner F. (2016). Assessment of cochlear trauma during cochlear implantation using electrocochleography and cone beam computed tomography. Otol. Neurotol. 37, 446–453. doi: 10.1097/MAO.0000000000000998, PMID: 26945317

[ref10] Dalbert A. Pfiffner F. Hoesli M. Koka K. Veraguth D. Roosli C.. (2018). Assessment of cochlear function during cochlear implantation by extra-and intracochlear electrocochleography. Front. Neurosci. 12:18. doi: 10.3389/fnins.2018.00018, PMID: 29434534 PMC5790789

[ref11] Dalbert A. Pfiffner F. Röösli C. Thoele K. Sim J. H. Gerig R.. (2015). Extra-and intracochlear electrocochleography in cochlear implant recipients. Audiol. Neurotol. 20, 339–348. doi: 10.1159/000438742, PMID: 26340649

[ref12] Dalbert A. Sijgers L. Grosse J. Veraguth D. Roosli C. Huber A.. (2021). Simultaneous intra-and extracochlear electrocochleography during electrode insertion. Ear Hear. 42, 414–424. doi: 10.1097/AUD.0000000000000935, PMID: 32826509

[ref13] Eggermont J. J. (1974). Basic principles for electrocochleography. Acta Otolaryngol. 77, 7–16. doi: 10.1080/16512251.1974.11675742, PMID: 4525558

[ref14] Fitzpatrick D. C. Campbell A. Choudhury B. Dillon M. Forgues M. Buchman C. A.. (2014). Round window electrocochleography just prior to cochlear implantation: relationship to word recognition outcomes in adults. Otol. Neurotol. 35, 64–71. doi: 10.1097/MAO.0000000000000219, PMID: 24317211 PMC4447311

[ref15] Forgues M. Koehn H. A. Dunnon A. K. Pulver S. H. Buchman C. A. Adunka O. F.. (2014). Distinguishing hair cell from neural potentials recorded at the round window. J. Neurophysiol. 111, 580–593. doi: 10.1152/jn.00446.2013, PMID: 24133227 PMC3921406

[ref16] Fröhlich M. Deutz J. Wangenheim M. Rau T. S. Kral A. Lenarz T.. (2024). The role of pressure and friction forces in automated insertion of Cochlear implants. Front. Neurol. 15:1430694. doi: 10.3389/fneur.2024.1430694 39170077 PMC11337231

[ref17] Fröhlich M. Schurzig D. Rau T. S. Lenarz T. (2024). On the interdependence of insertion forces, insertion speed, and lubrication: aspects to consider when testing cochlear implant electrodes. PLoS One 19:e0295121. doi: 10.1371/journal.pone.0295121, PMID: 38266033 PMC10807833

[ref18] Gantz B. J. Dunn C. C. Oleson J. Hansen M. R. (2018). Acoustic plus electric speech processing: long-term results. Laryngoscope 128, 473–481. doi: 10.1002/lary.26669, PMID: 28543270 PMC5700847

[ref19] Gantz B. J. Turner C. Gfeller K. E. Lowder M. W. (2005). Preservation of hearing in cochlear implant surgery: advantages of combined electrical and acoustical speech processing. Laryngoscope 115, 796–802. doi: 10.1097/01.MLG.0000157695.07536.D2, PMID: 15867642

[ref20] Giardina C. K. Brown K. D. Adunka O. F. Buchman C. A. Hutson K. A. Pillsbury H. C.. (2019). Intracochlear electrocochleography: response patterns during cochlear implantation and hearing preservation. Ear Hear. 40, 833–848. doi: 10.1097/AUD.0000000000000659, PMID: 30335669 PMC6534483

[ref21] Greisiger R. Bester C. Sørensen T. Korslund H. Bunne M. O'Leary S.. (2024). Intraoperative measured electrocochleography and fluoroscopy video to detect cochlea trauma. Otol. Neurotol. 45, 36–45. doi: 10.1097/MAO.0000000000004055, PMID: 38085760

[ref22] Haumann S. Imsiecke M. Bauernfeind G. Büchner A. Helmstaedter V. Lenarz T.. (2019). Monitoring of the inner ear function during and after cochlear implant insertion using electrocochleography. Trends Hear. 23:2331216519833567. doi: 10.1177/2331216519833567, PMID: 30909815 PMC6435875

[ref23] Haumann S. Mynarek M. Maier H. Helmstaedter V. Büchner A. Lenarz T.. (2024). Does intraoperative extracochlear electrocochleography correlate with postoperative audiometric hearing thresholds in cochlea implant surgery – a retrospective analysis on Cochlear monitoring. Trends Hear. 28, 1–22. doi: 10.1177/23312165241252240, PMID: 38715410 PMC11080760

[ref24] Haumann S. Timm M. E. Büchner A. Lenarz T. Salcher R. B. (2024). Intracochlear recording of electrocochleography during and after cochlear implant insertion dependent on the location in the cochlea. Trends Hear. 28, 1–16. doi: 10.1177/23312165241248973, PMID: 38717441 PMC11080744

[ref25] Helbig S. Van de Heyning P. Kiefer J. Baumann U. Kleine-Punte A. Brockmeier H.. (2011). Combined electric acoustic stimulation with the PULSARCI100 implant system using the FLEXEAS electrode array. Acta Otolaryngol. 131, 585–595. doi: 10.3109/00016489.2010.544327, PMID: 21281057

[ref26] Helpard L. Li H. Rohani S. A. Zhu N. Rask-Andersen H. Agrawal S.. (2021). An approach for individualized cochlear frequency mapping determined from 3D synchrotron radiation phase-contrast imaging. IEEE Trans. Biomed. Eng. 68, 3602–3611. doi: 10.1109/TBME.2021.3080116, PMID: 33983877

[ref27] Honrubia V. Ward P. H. (1968). Longitudinal distribution of the cochlear microphonics inside the cochlear duct (guinea pig). J. Acoust. Soc. Am. 44, 951–958. doi: 10.1121/1.1911234, PMID: 5683661

[ref28] Incerti P. V. Ching T. Y. Cowan R. (2013). A systematic review of electric-acoustic stimulation: device fitting ranges, outcomes, and clinical fitting practices. Trends Amplif. 17, 3–26. doi: 10.1177/1084713813480857, PMID: 23539259 PMC4040864

[ref29] James C. Albegger K. Battmer R. Burdo S. Deggouj N. Deguine O. (2005). Preservation of residual hearing with cochlear implantation: how and why. Acta Otolaryngol. 125, 481–491. doi: 10.1080/00016480510026197, PMID: 16092537

[ref30] Kim J.-S. (2020). Electrocochleography in Cochlear implant users with residual acoustic hearing: a systematic review. Int. J. Environ. Res. Public Health 17:7043. doi: 10.3390/ijerph17197043, PMID: 32993065 PMC7579537

[ref31] Kim J.-S. (2024). Clinical applications of intracochlear electrocochleography in cochlear implant users with residual acoustic hearing. J. Audiol. Otol. 28, 100–106. doi: 10.7874/jao.2024.00129, PMID: 38695055 PMC11065546

[ref32] Koka K. Riggs W. J. Dwyer R. Holder J. T. Noble J. H. Dawant B. M. (2018). Intra-cochlear electrocochleography during cochear implant electrode insertion is predictive of final scalar location. Otol. Neurotol. 39:e654, –e659. doi: 10.1097/MAO.0000000000001906, PMID: 30113557 PMC6097527

[ref33] Koka K. Saoji A. A. Litvak L. M. (2017). Electrocochleography in cochlear implant recipients with residual hearing: comparison with audiometric thresholds. Ear Hear. 38, e161–e167. doi: 10.1097/AUD.0000000000000385, PMID: 27879487

[ref34] Krüger B. Büchner A. Lenarz T. Nogueira W. (2020). Amplitude growth of intracochlear electrocochleography in cochlear implant users with residual hearing. J. Acoust. Soc. Am. 147, 1147–1162. doi: 10.1121/10.0000744, PMID: 32113296

[ref35] Lenarz T. Büchner A. Illg A. (2022). Cochlear implantation: concept, results outcomes and quality of life. Laryngo-Rhino-Otologie 101, S36–S78. doi: 10.1055/a-1731-9321, PMID: 35605612

[ref36] Lenarz T. Stöver T. Buechner A. Lesinski-Schiedat A. Patrick J. Pesch J. (2009). Hearing conservation surgery using the hybrid-L electrode. Audiol. Neurotol. 14, 22–31. doi: 10.1159/000206492 19390172

[ref37] Lenarz T. Timm M. E. Salcher R. Büchner A. (2019). Individual hearing preservation cochlear implantation using the concept of partial insertion. Otol. Neurotol. 40, e326–e335. doi: 10.1097/MAO.0000000000002127, PMID: 30741914

[ref38] O’Leary S. Mylanus E. Venail F. Lenarz T. Birman C. Di Lella F. (2023). Monitoring cochlear health with intracochlear electrocochleography during cochlear implantation: findings from an international clinical investigation. Ear Hear. 44, 358–370. doi: 10.1097/AUD.0000000000001288, PMID: 36395515 PMC9957964

[ref39] O'Malley J. T. Wu P.-Z. Kaur C. Gantz B. J. Hansen M. R. Quesnel A. M.. (2024). Delayed hearing loss after cochlear implantation: re-evaluating the role of hair cell degeneration. Hear. Res. 447:109024. doi: 10.1016/j.heares.2024.109024, PMID: 38735179 PMC11134194

[ref40] Panario J. Bester C. O’Leary S. (2024). Predicting postoperative speech perception and audiometric thresholds using intracochlear electrocochleography in cochlear implant recipients. Ear Hear. 149, 1120–1129. doi: 10.1001/jamaoto.2023.2988, PMID: 38816899

[ref41] Quesnel A. M. Nakajima H. H. Rosowski J. J. Hansen M. R. Gantz B. J. Nadol J. B. Jr. (2016). Delayed loss of hearing after hearing preservation cochlear implantation: human temporal bone pathology and implications for etiology. Hear. Res. 333, 225–234. doi: 10.1016/j.heares.2015.08.018, PMID: 26341474 PMC4775460

[ref42] Radeloff A. Shehata-Dieler W. Scherzed A. Rak K. Harnisch W. Hagen R.. (2012). Intraoperative monitoring using cochlear microphonics in cochlear implant patients with residual hearing. Otol. Neurotol. 33, 348–354. doi: 10.1097/MAO.0b013e318248ea86, PMID: 22377649

[ref43] Robles L. Ruggero M. A. (2001). Mechanics of the mammalian cochlea. Physiol. Rev. 81, 1305–1352. doi: 10.1152/physrev.2001.81.3.1305, PMID: 11427697 PMC3590856

[ref44] Roland J. T. Jr. Gantz B. J. Waltzman S. B. Parkinson A. J. (2018). Long-term outcomes of cochlear implantation in patients with high-frequency hearing loss. Laryngoscope 128, 1939–1945. doi: 10.1002/lary.27073, PMID: 29330858 PMC6792393

[ref45] Saoji A. A. Graham M. K. Adkins W. J. Koka K. Carlson M. L. Neff B. A.. (2023). Multi-frequency electrocochleography and electrode scan to identify electrode insertion trauma during cochlear implantation. Brain Sci. 13:330. doi: 10.3390/brainsci13020330, PMID: 36831873 PMC9954676

[ref46] Saoji A. A. Patel N. S. Carlson M. L. Neff B. A. Koka K. Tarigoppula V. S.. (2019). Multi-frequency electrocochleography measurements can be used to monitor and optimize electrode placement during cochlear implant surgery. Otol. Neurotol. 40, 1287–1291. doi: 10.1097/MAO.0000000000002406, PMID: 31644474

[ref47] Schurzig D. Repp F. Timm M. E. Batsoulis C. Lenarz T. Kral A. (2023). Virtual cochlear implantation for personalized rehabilitation of profound hearing loss. Hear. Res. 429:108687. doi: 10.1016/j.heares.2022.108687, PMID: 36638762

[ref48] Schurzig D. Timm M. E. Batsoulis C. John S. Lenarz T. (2018). Analysis of different approaches for clinical cochlear coverage evaluation after cochlear implantation. Otol. Neurotol. 39, e642–e650. doi: 10.1097/MAO.0000000000001904, PMID: 30015749

[ref49] Seyyedi M. Nadol J. B. Jr. (2014). Intracochlear inflammatory response to cochlear implant electrodes in the human. Otol. Neurotol. 35, 1545–1551. doi: 10.1097/MAO.0000000000000540, PMID: 25122600 PMC4165780

[ref50] Sijgers L. Pfiffner F. Grosse J. Dillier N. Koka K. Röösli C.. (2021). Simultaneous intra-and extracochlear electrocochleography during cochlear implantation to enhance response interpretation. Trends Hear. 25:2331216521990594. doi: 10.1177/2331216521990594, PMID: 33710919 PMC7958165

[ref51] Sijgers L. Sorensen T. Soulby A. Boyle P. Dalbert A. Röösli C. (2023). Classification of acoustic hearing preservation after cochlear implantation using electrocochleography. Trends Hear. 27:23312165231220997. doi: 10.1177/23312165231220997, PMID: 38105510 PMC10729624

[ref52] Snyder R. L. Schreiner C. E. (1984). The auditory neurophonic: basic properties. Hear. Res. 15, 261–280. doi: 10.1016/0378-5955(84)90033-9, PMID: 6501114

[ref53] Suhling M.-C. Majdani O. Salcher R. Leifholz M. Büchner A. Lesinski-Schiedat A.. (2016). The impact of electrode array length on hearing preservation in cochlear implantation. Otol. Neurotol. 37, 1006–1015. doi: 10.1097/MAO.0000000000001110, PMID: 27309713

[ref54] Tan E. Bester C. Collins A. Razmovski T. O'Leary S. (2024). Four-point impedance: a potential biomarker for residual hearing after cochlear implantation. Otol. Neurotol. 45, e315–e321. doi: 10.1097/MAO.0000000000004153, PMID: 38478410

[ref55] Timm M. E. Majdani O. Weller T. Windeler M. Lenarz T. Büchner A.. (2018). Patient specific selection of lateral wall cochlear implant electrodes based on anatomical indication ranges. PLoS One 13:e0206435. doi: 10.1371/journal.pone.0206435, PMID: 30365565 PMC6203394

[ref56] Von Ilberg C. A. Baumann U. Kiefer J. Tillein J. Adunka O. F. (2011). Electric-acoustic stimulation of the auditory system: a review of the first decade. Audiol. Neurotol. 16, 1–30. doi: 10.1159/000327765, PMID: 21606646

[ref57] Walia A. Shew M. A. Lee D. S. Lefler S. M. Kallogjeri D. Wick C. C. (2022). Promontory Electrocochleography recordings to predict speech-perception performance in Cochlear implant recipients. Otol. Neurotol. 43, 915–923. doi: 10.1097/MAO.0000000000003628, PMID: 35861658 PMC9621328

[ref58] Walia A. Shew M. A. Varghese J. Lefler S. M. Bhat A. Ortmann A. J.. (2024). Electrocochleography-based tonotopic map: II. Frequency-to-place mismatch impacts speech-perception outcomes in cochlear implant recipients. Ear Hear. 45, 1406–1417. doi: 10.1097/AUD.0000000000001528, PMID: 38880958 PMC11493529

[ref59] Würfel W. Lanfermann H. Lenarz T. Majdani O. (2014). Cochlear length determination using cone beam computed tomography in a clinical setting. Hear. Res. 316, 65–72. doi: 10.1016/j.heares.2014.07.013, PMID: 25124151

